# A perceptual account of symbolic reasoning

**DOI:** 10.3389/fpsyg.2014.00275

**Published:** 2014-04-21

**Authors:** David Landy, Colin Allen, Carlos Zednik

**Affiliations:** ^1^Psychological and Brain Science/Cognitive Science, Indiana UniversityBloomington, IN, USA; ^2^History and Philosophy of Science/Cognitive Science, Indiana UniversityBloomington, IN, USA; ^3^Institute of Cognitive Science, University of OsnabrückOsnabrück, Germany

**Keywords:** human reasoning, formal logic, mathematics, embodied cognition, perception

## Abstract

People can be taught to manipulate symbols according to formal mathematical and logical rules. Cognitive scientists have traditionally viewed this capacity—the capacity for *symbolic reasoning*—as grounded in the ability to internally represent numbers, logical relationships, and mathematical rules in an abstract, amodal fashion. We present an alternative view, portraying symbolic reasoning as a special kind of embodied reasoning in which arithmetic and logical formulae, externally represented as notations, serve as targets for powerful perceptual and sensorimotor systems. Although symbolic reasoning often conforms to abstract mathematical principles, it is typically implemented by perceptual and sensorimotor engagement with concrete environmental structures.

## Introduction

How do people reason arithmetically, algebraically, and logically? One well-known answer to this question holds that the human mind trades in inner symbols that amodally represent abstract arithmetic, algebraic, and logical propositions, and manipulates these symbols according to internally represented mathematical and logical rules. On this traditional view, the “inner” takes precedence over the “outer”: notations on paper, computer screens, and classroom blackboards are involved in mathematical problem-solving only insofar as they are “translated” into corresponding mental structures and processes.

**Figure 1:**
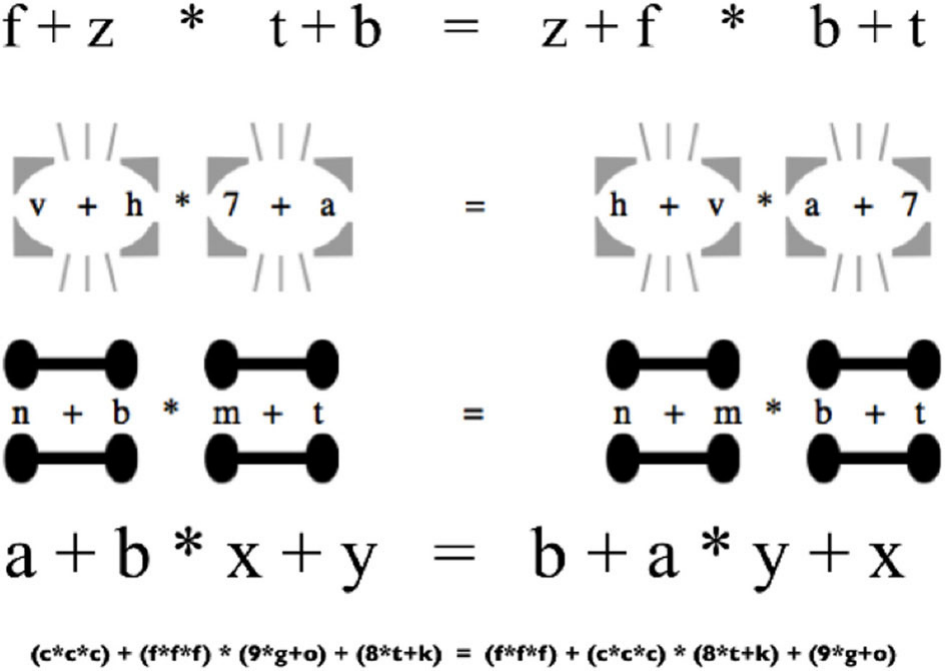
**Some of the formats employed by Landy and Goldstone (2007a)**. Visual cues such as added spacing, lines, and circles influence the application of perceptual grouping mechanisms, influencing the capacity for symbolic reasoning.

Suppose you hold such a traditional view, but then learn that stray marks and subtle changes in spacing can lead otherwise competent students of algebra to “forget” a basic rule such as operator precedence. Several recent experiments have demonstrated just this sort of influence of visual structure on algebraic performance. One example comes from Landy and Goldstone (2007a), who gave college undergraduates simple algebraic forms, such as “*a* + *b* ∗ *c* + *d* = *c* + *d* ∗ *a* + *b*,” and asked them to decide whether or not the given symbols described a valid equation (see Figure 1). Because the expressions contained both additions and multiplications, determining their validity required respecting the order of operations, which stipulates that multiplications precede additions. By creating artificial visual groups (e.g., by manipulating the physical spacing of equations, or by introducing shapes into the surrounding context as depicted in Figure 1), participants' performance could be predictably manipulated: validity-judgments were more likely to be correct if visual groupings were in line with valid operator precedence. Nor is this pattern restricted to algebraic validity. Related research has indicated that spatial layout impacts application of the order of operations rules when calculating (Kirshner, 1989; Landy and Goldstone, 2010), when creating story problems (Jiang et al., in press), and when working in programming languages such as Python (Hansen et al., unpublished manuscript).

How might you interpret this sort of behavioral pattern? You could chalk failure to respect operator precedence, for example, up to performance error, and remain committed to the thesis that the underlying mathematical competence is largely independent of the way notational structures are perceived and physically manipulated. Alternatively, you could wonder whether competence with operator precedence depends non-trivially on the perceptual and sensorimotor mechanisms that target those external notations. To what extent might these mechanisms be responsible not just for our mathematical mistakes, but also for our successes?

The ability to follow operator-precedence rules is just one manifestation of the capacity for *symbolic reasoning*: the capacity to manipulate arbitrary symbolic tokens according to abstract mathematical and logical rules. In what follows, we propose an account of symbolic reasoning according to which perception, manipulation, and perceptual imagination lie at the heart of mathematical and logical competence. Rather than rely on amodally represented rules, symbolic reasoners make their mathematical judgments using perceptual processes that have no obvious link to the following of formal mathematical rules. Instead, we identify the capacity for symbolic reasoning with the ability to perceptually group, detect symmetry in, and otherwise perceptually organize symbolic notations as they are experienced in the environment. On this view, the kinds of behavioral patterns described above are typical: not only does written format impact the legibility of symbols, it also impacts the application of well-known rules. When notational expressions afford active manipulation, symbolic reasoning is often accomplished by physically interacting with those notations. In contrast, when notations do not afford physical manipulation or perceptual processing, symbolic reasoning may involve processes of visual, aural, and even tactile imagination. Although symbolic reasoning can therefore become “internalized,” it remains rooted in mechanisms close to the sensorimotor periphery.

Although we will emphasize the kinds of algebra, arithmetic, and logic that are typically learned in high school, our view also potentially explains the activities of advanced mathematicians—especially those that involve representational structures like graphs and diagrams. Our major goal, therefore, is to provide a novel and unified account of both successful and unsuccessful episodes of symbolic reasoning, with an eye toward providing an account of mathematical reasoning in general. Before turning to our own account, however, we begin with a brief outline of some more traditional views.

## Extant accounts of symbolic reasoning

### Computationalism and semantic processing: translational accounts of symbolic reasoning

Two prominent accounts of symbolic reasoning can be introduced via an analogy from the classroom. Consider the different ways in which students might be taught to think about the following syllogism:

All dogs are mammals;
All mammals are animals;
Therefore, all dogs are animals.

On one hand, students can think about such problems *syntactically*, as a specific instance of the more general logical form “All *X*s are *Y*s; All *Y*s are *Z*s; Therefore, all *X*s are *Z*s.” On the other hand, they might think about them *semantically*—as relations between subsets, for example. In an analogous fashion, two prominent scientific attempts to explain how students are able to solve symbolic reasoning problems can be distinguished according to their emphasis on syntactic or semantic properties.

Analogous to the syntactic approach above, *computationalism* holds that the capacity for symbolic reasoning is carried out by mental processes of syntactic rule-based symbol-manipulation. In its canonical form, these processes take place in a general-purpose “central reasoning system” that is functionally encapsulated from dedicated and modality-specific sensorimotor “modules” (Fodor, 1983; Sloman, 1996; Pylyshyn, 1999; Anderson, 2007). Although other versions of computationalism do not posit a strict distinction between central and sensorimotor processing, they do generally assume that sensorimotor processing can be safely “abstracted away” (e.g., Kemp et al., 2008; Perfors et al., 2011). On all computationalist accounts, when an individual is confronted with a symbolic reasoning task such as a natural-language “word problem” or a formal reasoning problem expressed in the notational formalisms of algebra, calculus, and logic, the perception of notations in the environment causes a tokening of equivalent symbols and expressions of “Mentalese” (Fodor, 1975). These mental symbols and expressions are then operated on by syntactic rules that instantiate mathematical and logical principles, and that are typically assumed to take the form of productions, laws, or probabilistic causal structures (Newell and Simon, 1976; Sloman, 1996; Anderson, 2007). Once a solution is computed, it is converted back into a publicly observable (i.e., written or spoken) linguistic or notational formalism.

An influential alternative to computationalism is analogous to the semantic approach to the syllogism above: the heterogeneous family of *semantic processing* accounts, according to which symbolic reasoning is carried out by systems that interpret and represent meaningful mathematical and logical relations. Accounts of this type differ according to the particular representational formats they posit, ranging from amodal or generically spatial “mental models” (Johnson-Laird et al., 1992), to rich perceptual and sensorimotor “simulations” of specific objects and scenes (Barsalou, 1999), and even to indirect “conceptual metaphors” that drive people's intuitions and conclusions about a specific mathematical problem (Lakoff and Nuñez, 2000). What distinguishes these accounts from computationalism is the idea that symbolic reasoning occurs not on the basis of syntactic rules, but on the basis of meaningful interpretations of a particular mathematical or logical task domain. For example, Lakoff and Nuñez argue that real-number concepts are derived from experiences with physical lengths, and that the capacity for simple arithmetic arises from an innate ability to estimate and compare such lengths. On Johnson-Laird's “mental models” account, symbolic reasoning problems are solved by “inspecting” a mental model of the problem: the validity of “*a* & *b* ∴ *b*” can be determined by recognizing that “*b*” is a component of the model for “*a* & *b*.” In much the same way, Barsalou's “perceptual symbol systems” account suggests that logical expressions are interpreted by mentally simulating concrete scenarios to which the expression applies: a scene that includes both an apple and an orange includes an orange.

Despite their differences, computationalist, and semantic processing accounts share the assumption that processes of perception and action play a relatively limited role in the process of symbolic reasoning. Although both accounts acknowledge that the perception of notations is important for the construction of internal representations, they also assume that once such representations have been constructed, the physical notations that express the original mathematical or logical problem may be ignored or altogether discarded until a solution is communicated. Notably, this even applies to accounts which, like Barsalou's, posit a special role for sensorimotor representations in general, yet attribute a curiously limited role to sensorimotor representations of the notations that are actually perceived while a symbolic reasoning task is being performed. In general, computationalist and sematic processing accounts are alike in being essentially *translational*: they suppose that processes of perception and action do little other than mediate between notational structures in the external environment and the internal structures and processes in which symbolic reasoning *really* occurs.

It is worth elaborating on this translational aspect. The capacity for symbolic reasoning is expressed behaviorally by converting an input representation of a mathematics or logic problem into an output representation of a corresponding solution. Initially, the problem is represented in a public language, either as a natural-language “word problem”, or in the special notational systems designed for algebra, calculus, and logic. Eventually, this problem representation is converted into a written or spoken solution. But exactly how does this conversion occur? Like many other kinds of problem solving, the process of symbolic reasoning can be seen as a chain of transformations that links input and output representations, each of which changes its format and/or semantic structure. Some transformations, such “*a* and *b*” to “*a* & *b*,” involve a change in format without a change in semantic structure. In contrast, transformations such as “~(~*a* ∨ ~*b*) ∴ *b*” to “*a* & *b* ∴ *b*” involve changes in format *and* semantic structure: the resulting representation is a simplification of the original problem.

Computationalist and semantic processing accounts of symbolic reasoning are equally translational because they both assume that problem representations are passed from a perceptual apparatus to an internal processing system in a form that is no simpler than the external (notational or linguistic) problem representation. That is, they assume that all transformations that involve changes in semantic structure take place “internally,” over Mentalese expressions, mental models, metaphors or simulations, and that sensorimotor interactions with physical notations involve (at most) a change in representational format. On these accounts, when a subject is asked to evaluate a formal expression such as “~(~*a* ∨ ~*b*) ∴ *b*,” a mental representation of that expression must be constructed before it can be simplified to “*a* & *b* ∴ *b*.” Similarly, notational variants of one-and-the-same proposition—e.g., “*All Fs are Gs*,” “(*x*)(*Fx* → *Gx*),” and “∀*x*[*Fx* ⊃ *Gx*]” will be converted into one-and-the-same Mentalese expression, mental model, metaphor or simulation. In general, therefore, computationalist and semantic processing accounts of symbolic reasoning rely equally on the assumption that the principal role of sensorimotor processes—the processes that govern the perception of and physical interaction with public symbols and expressions—is simply to provide inputs to and carry outputs from those internal structures and processes that are ultimately responsible for performing all substantial steps in a mathematical or logical problem solving chain.

### Toward a constitutive account: the cyborg view

Translational accounts of symbolic reasoning can be distinguished from *constitutive* accounts, in which sensorimotor mechanisms are not merely part of the causal chain that links external notations to internal representations, but are crucially involved in transforming the problem representation into one that has a simplified semantic structure. Recall that on the translationist view, mental resources can be divided into those that “translate” the outer situation into a generally isomorphic inner representation, and those that act on that representation to solve the problem. On a constitutive account, sensorimotor mechanisms not only translate the problem, they are involved in the transformations that substantively solve it. One prominent view that can be associated with such a constitutive approach might, to borrow Andy Clark's terminology, be called the *cyborg view* of symbolic reasoning (Clark, 2003). Grounded on recent work in the area of “situated cognition,” the cyborg view holds that notations constitute external technological artifacts that “scaffold” the biological processes involved in symbolic reasoning (Clark, 1997, 1998, 2006; Menary, 2007; Sutton, 2010). This “scaffolding” is typically achieved by notations that permit the extraneural storing, inspection, deletion and manipulation of information in a way that facilitates the execution of symbolic reasoning tasks, and has positive effects on the speed and accuracy with which these tasks can be performed as well as their potential complexity. To cite a well-known example, “carrying” a digit during a complex multiplication task by writing it on a piece of paper, adding it to the result and then crossing it out obviates the need to store and manipulate that digit in biological memory, thereby freeing up valuable cognitive resources, minimizing possible error from misremembering, and permitting the multiplication of extremely large values. One way of explaining the cognitive benefit of such “scaffolding” is to view notations as constitutive parts of integrated, boundary-crossing symbolic reasoning systems: When computing “123 × 89”, “carrying” the tens digit of the temporary product “3 × 9” and adding it to the units digit of “2 × 9” transforms the original complex multiplication problem into a series of simpler multiplication and addition problems that can easily be done in the head. Thus, the active manipulation of physical notations plays the role of “guiding” the human biological machinery through an abstract mathematical problem space—one that may far exceed the space of otherwise solvable problems.

While emphasizing the ways in which notations are acted upon, however, proponents of the cyborg view rarely consider how such notations are perceived. Sometimes, this neglect is intentional, as when the utility of cognitive artifacts is explained by stating that they become assimilated into a “body schema” in which “sensorimotor capacities function without… the necessity of perceptual monitoring” (Gallagher, 2005, p. 25). At other times, this neglect seems to be unintended, however, and subject to corrective elaboration. For example, although Andy Clark (1998, p. 168) argues that the human ability to deploy and manipulate notations in symbolic reasoning tasks “involves the use of the same old (essentially pattern-completing) resources to model the special kinds of behavior observed in the public [notational] world,” it remains unclear exactly *which* pattern-completing resources are in play, and what kinds of patterns they complete. In general, therefore, although cyborg theorists have shown quite successfully that notations can be constitutively involved in symbolic reasoning, and have made great strides in cataloguing the kinds of bodily interactions that lead to cognitive success, few specific details have emerged regarding the relevant perceptual processes that facilitate these interactions, as well as the physical characteristics that determine when and why a particular notation is cognitively beneficial.

Consider how such details might explain the influence of visual structure on algorithmic reasoning discussed earlier. Order of operations behavior need not be implemented in a set of high-level productions or in a collection of explicit memorized rules, but also need not be determined by active manipulations of physical notations. Instead, such behavior might largely depend on visual processes that segment the scene into parts, wholes, and groups. One possibility is that because the algebraic system tends to align spatial structure and precedence rules, perceptual grouping processes acquire biases compatible with those rules (Kirshner and Awtry, 2004); another is that because proofs tend to maintain tightly bound structures, leading to increased statistical regularity in high precedence operations, experience with algebraic derivations modifies perceptual organization. Other regular cultural cues have long been known to impact grouping (Wertheimer, 1923/1938). By extending the cyborg view's emphasis on environmental interaction with a detailed understanding of perceptual processing, a theoretical framework might be developed that accounts for the effect of aligning visual grouping and syntactic binding discussed earlier (see Figure 1), but that may also explain many other episodes of formally correct and incorrect symbolic reasoning.

In what follows, we articulate a constitutive account of symbolic reasoning, *Perceptual Manipulations Theory*, that seeks to elaborate on the cyborg view in exactly this way. While accommodating the cyborg view's emphasis on the active manipulation of physical notations, Perceptual Manipulations Theory additionally emphasizes the perceptual processes that facilitate and govern such manipulations, as well as the physical characteristics of particularly successful (and unsuccessful) notational formalisms. On our view, the way in which physical notations are perceived is at least as important as the way in which they are actively manipulated.

## Perceptual manipulations theory

### The theory

Perceptual Manipulations Theory (PMT) goes further than the cyborg account in emphasizing the perceptual nature of symbolic reasoning. External symbolic notations need not be translated into internal representational structures, but neither does all mathematical reasoning occur by manipulating perceived notations on paper. Rather, complex visual and auditory processes such as affordance learning, perceptual pattern-matching and perceptual grouping of notational structures produce simplified representations of the mathematical problem, simplifying the task faced by the rest of the symbolic reasoning system. Perceptual processes exploit the typically well-designed features of physical notations to automatically reduce and simplify difficult, routine formal chores, and so are themselves constitutively involved in the capacity for symbolic reasoning. Moreover, if a particular symbolic reasoning problem cannot be solved by perceptual processing and active manipulation of physical notations alone, subjects often invoke detail-rich sensorimotor representations that closely resemble the physical notations in which that problem was originally encountered. On our view, therefore, much of the capacity for symbolic reasoning is implemented as the perception, manipulation and modal and cross-modal representation of externally perceived notations.

The neural processes that PMT takes to be involved in symbolic reasoning almost never have as their primary function the implementation of amodally represented rules or models. Instead, they include sensorimotor systems for visual grouping and perceptual organization, object recognition, object tracking and symmetry detection, among others. Although skills such as object-recognition may appear quintessentially “cognitive” to some, we treat them as sensorimotor capacities to highlight the fact that, rather than apply to abstract mathematical or logical entities, they apply directly to the physical properties of notations in the environment such as shape, relative spacing and position. Indeed, insofar as most mathematical and logical notations are well-designed, these properties are frequently suggestive of how they ought to be manipulated, thus promoting formally valid “symbol-pushing”. For example, the fact that the multiplicands in “*xy* + *z*” are closer to one another than to the additive term can be understood as a manifestation of the order-of-operations rule that multiplication is to be performed before addition—a manifestation that is immediately recognized by mechanisms of perceptual grouping (see section Evidence for Perceptual Manipulations Theory). Notably, such sensorimotor competences are often more robust than the formal systems to which they are applied: while a formula such as “(((P→((Q&R)” would be rejected by a machine following strict well-formedness rules, even beginning logic students interpret it as a conditional, and must be explicitly trained by pedagogues with ulterior motives to focus on a narrower set of structural elements. As we discuss in greater detail below, a wide range of (correct *and* incorrect) mathematical behavior can be attributed to the way the perceived details of formal notations “interlock” with domain-general sensorimotor capacities.

Perceptual Manipulations Theory suggests that most symbolic reasoning emerges from the ways in which notational formalisms are perceived and manipulated. Nevertheless, direct sensorimotor processing of physical stimuli is augmented by the capacity to imagine and manipulate mental representations of notational markings. Faculties of spatial reasoning, mental transformation, referential symbolism and a rich set of capacities for acquiring and imagining physical behaviors such as walking, pointing, writing, and erasing can all be used to internally reproduce the actual perceived details of physical notations and to mentally manipulate them in ways that resemble physical actions. Insofar as our account emphasizes perceptual representations of formal notations and imagined notation-manipulations, it can be contrasted with Barsalou's perceptual symbol systems account, in which “people often construct non-formal simulations to solve formal problems” (Barsalou, 1999, 606). Moreover, our emphasis differs from standard “conceptual metaphor” accounts, which suggest that formal reasoners rely on a “semantic backdrop” of embodied experiences and sensorimotor capacities to interpret abstract mathematical concepts. Our account is probably closest to one articulated by Dörfler (2002), who like us emphasizes the importance of treating elements of notational systems as physical objects rather than as meaning-carrying symbols.

Although there are clear differences between PMT and other accounts of symbolic reasoning, our view incorporates elements from many of them—albeit with a greater emphasis on perception. For illustration, consider a student already competent in logic now learning set theory. The perceivable physical similarities of ∩ and ∪ to ∧ and ∨, including the up-down symmetry between each pair, serve as a *perceptual*, rather than conceptual, metaphor. To see how this metaphor may be applied, consider the duality principle that

$$\bar{A\cup B}=\bar{A}\cap \bar{B}$$

which bears a striking visual similarity to De Morgan's law,

$$\bar{P\vee Q}\equiv \bar{P}\wedge \bar{Q}$$

This visual similarity is partially a result of common symbology, including the use of capital letters for elements, the use of horizontal lines for equality, the use of bars for negation, and the above-mentioned use of similar shapes for basic operations. Partially, though, the similarity results from the arrangement of these parts—if one is written in prefix notation, for instance, the similarity is markedly decreased (it is beyond the scope of this work to attempt a general definition of similarity; for a review, see Goldstone and Son, 2005). For a student learning a new formal system, these notational similarities ground the transformations typical to set theory by mapping them onto the more familiar domain of logic, facilitating the application of similar principles and ideas, and licensing particular manipulations, sometimes even prior to obtaining a rich understanding of the conceptual issues involved. To the degree that these inferences are licensed, learning may be facilitated. Although the relevant perceptual and sensorimotor processes are modality-specific, when mathematical notations are well-designed, human mathematical competence can be incredibly flexible: radically different mathematical and logical propositions can be treated in similar formal ways because of similarities in the way in which they are physically manifested as notations. Of course, it is not always or often the case that capturing visual and semantic regularities across domains is the explicit goal of mathematicians introducing notation (though see Smaill, 2012, for one apparent case). We predict, however, that when there are significant visual similarities in notations used across domains, people will tend to import assumptions from a well-understood domain into a novel one.

Perceptual Manipulations Theory also posits a novel psychological role for much-discussed magnitude- and quantity-detection systems. Visual quantity (e.g., the number of blocks, dots, or sheep presented in a drawing or on a computer screen) is often thought to be directly represented by an evolved “number system” dedicated to amodal magnitude representation (Gelman and Gallistel, 1978; Barth et al., 2003; Dehaene et al., 2004; Machery, 2007). It has been argued that such quantity-sensitive mechanisms provide the basic representational vehicles over which formal mathematical reasoning occurs (Gallistel et al., 2005; Spelke, 2005; Carey, 2009), but PMT holds a more textured view. Quantity-sensitive mechanisms certainly sometimes represent numbers. In symbolic reasoning tasks, however, a primary function of magnitude and quantity-detection systems is to enable reasoners to track magnitude and quantity properties of notational formalisms. For example, when dealing with large numbers such as “ 3,000,000,” magnitude-detection plays a role in keeping track of the number of digits (Hinrichs et al., 1982). Similarly, when teaching a rule such as the product rule captured by “*a*^5^*a*^3^ = *a*^8^,” a teacher may write something like “ (*aaaaa*) × (*aaa*) = (*aaaaaaaa*)” and let magnitude-detection (and explicit counting) systems do the rest. Thus, a significant portion of the verification process may be implemented by perceptual and sensorimotor skills and quantity-detection systems that process the notational formalism itself, without necessarily interpreting the notation's meaning.

The emphasis that PMT places on domain-general systems for perceptual processing and bodily interaction with physical systems of notations underscores the importance of the historical development of a common set of well-designed mathematical notations. Although historically the development of visual commonalities across notations may have been largely accidental, this development has served mathematics well, providing visual cues that allow the human perceptual and motor systems to effectively operate over them. One prediction of PMT is that when notations align perceptual and structural similarities, learning will be facilitated. Of course, when they misalign, as they sometimes do, learning is predicted to be impaired (Marquis, 1988 discusses several such cases). Still, better notation systems could yet be constructed in all branches of formal reasoning to take full advantage of visual cues that automatically “steer” the reasoner in the direction of formally valid solutions. In this way, the human capacity for symbolic reasoning winds up being ordinary, bodily situatedness in novel, artifactual sensorimotor space: the space of (well-designed!) notations.

### Evidence for perceptual manipulations theory

Most of the existing literature on symbolic reasoning has been developed using an implicitly or explicitly translational perspective. Although we do not believe that the current evidence is enough to completely dislodge this perspective, it does show that sensorimotor processing influences the capacity for symbolic reasoning in a number of interesting and surprising ways. The translational view easily accounts for cases in which individual symbols are more readily perceived based on external format. For example, blurring symbols will make them harder to perceive. Perceptual Manipulations Theory also predicts this sort of impact, but further predicts that perceived structures will affect the application of rules—since rules are presumed to be implemented via systems involved in perceiving that structure. In this section, we will review several empirical sources of evidence for the impact of visual structure on the implementation of formal rules. Although translational accounts may eventually be elaborated to accommodate this evidence, it is far more easily and naturally accommodated by accounts which, like PMT, attribute a constitutive role to perceptual processing.

Perceptual Manipulations Theory holds that skill with symbol systems is implemented in alignments between elements of external notations and perceptual and motor systems. Therefore, it predicts that the physical appearance of notations should strongly influence formal behavior. For example, it should be difficult to differentially respond to two similar-looking notational forms even if they are conceptually dissimilar. Substantial evidence suggests that this prediction holds. For example, Kirshner and Awtry (2004) show that the common mistake of confusing the valid rule regarding multiplication of two like terms by adding their exponents (*a*^*n*^ ∗ *a*^*m*^ = *a*^*n* + *m*^) with the visually similar but invalid rule regarding added terms (*a*^*n*^ + *a*^*m*^ = *a*^*n* + *m*^) can be avoided by teaching students a linguistic notation in which these equations no longer resemble one another. In the same way, common mistakes such as

$$\frac{a}{x}+\frac{b}{y}=\frac{a+b}{x+y}$$

can be prevented just by changing the notational format in which they are learned (see Marquis, 1988 for several examples of visual patterns in algebra). The frequency of these mistakes—as well as the fact that they can be prevented by switching notational formats—are hard to explain from a translational perspective in which perceived problems are converted into inner propositions or models, and in which formal dissimilarity ought to trump visual similarity. In contrast, they are quite easily explained from a perspective that attributes a constitutive role to perceptual processing. What appears to be happening is that students apply a very general maxim of perceptual pattern learning: if two things look similar, similar things can probably be done with them, and if they look different, they require different actions. Although this is not a formally valid way of reasoning over symbol systems (and indeed, often leads to the mistakes reported above), this general strategy may lead to correct solutions whenever visual similarity *does* mirror formal similarity (see also Cohen Kadosh, 2009). Indeed, such mirroring is widespread, and appears to be regularly exploited by reasoners. Consider the way algebraic notation aligns formal structure with perceptual grouping in the expression

$$\frac{a+b}{a+bc}.$$

Here, formal structure is mirrored in the visual grouping structure created both by the spacing (*b* and *c* are multiplied, then added to *a*) and by the physical demarcation of the horizontal line. Instead of applying abstract mathematical rules to process such expressions, Landy and Goldstone (2007a,b see also Kirshner, 1989) propose that reasoners leverage visual grouping strategies to directly segment such equations into multi-symbol visual chunks. To test this hypothesis, they investigated the way manipulations of visual groups affect participants' application of operator precedence rules. Maruyama et al. (2012) argue on the basis of fMRI and MEG evidence that mathematical expressions like these are parsed quickly by visual cortex, using mechanisms that are shared with non-mathematical spatial perception tasks.

Interestingly, perceptual processes play a role not only in the way notations are perceived, but also in the way they are created. By studying beginning logic students' physical arrangement of logical formulae in an online natural deduction tutoring system (Allen and Menzel, 2007), Landy and Goldstone (2007b) found statistically significant patterns of space-insertion consistent with the hypothesis that spaces are used to aid visual grouping within logical formulae. That is, reasoners not only exploit visual groups that are already present in the physical representation of a symbolic reasoning task, but also actively and endogenously reproduce such groups when they make it easier to find a solution. But why do reasoners insert such formally irrelevant features to their written notational formalisms? From a translational perspective, this question is difficult to answer: once a solution to a symbolic reasoning problem is computed, it merely needs to be translated into a public language, one in which the observed space-insertion patterns are formally irrelevant. From the perspective of PMT, however, it seems likely that such patterns either derive from the possibility that mathematical and logical equations are internally encoded in a perceptually-rich format in which details about spacing is retained, or from the utility of such patterns in computing intermediate solutions on paper by applying the same visual object-segmentation systems that were initially used to interpret the problem. Supporting the possibility that spatial structure plays a crucial role in the process of interpretation of equations, Jiang et al. (in press) report that subjects inventing story problems match the physical structure of provided equations.

The visual system is well-known to be particularly responsive to dynamic stimuli such as motion. This is reflected in the apparent relevance of motion and transformation in algebraic understanding of proofs. Nogueira de Lima and Tall (2007) documented that schoolchildren learning algebra often treat transformations such as

$$\begin{array}{l} x+b=y-m\\ x=y-m-b \end{array}$$

not as the repeated application of formal Euclidean axioms, but as “magic motion,” in which a term moves to the other side of the equation and “flips” sign. Landy and Goldstone (2009) suggest that this reference to motion is no mere metaphor. Subjects with significant training in calculus found it easier to solve problems of this form when an irrelevant field of background dots moved in the same direction as the variables, than when the dots moved in the contrary direction.

One suggestion of PMT is that mathematical concepts may be encoded using multiple strategies, and that perceptual-motor strategies may emerge over the process of using a symbol system. As an example, Varma and Schwartz (2011) examine the case of negative number acquisition, and in particular the acquisition of processes allowing the comparison of positive and negative numbers. Initially, learners are faster at comparing numbers that are close together when one is positive and the other negative—a reversal of the usual *distance effect* that holds with positive numbers (Moyer and Landauer, 1967)—but one that is consistent with a rule-based strategy involving comparing signs. More expert learners show a typical size effect, so that numbers that are ‘far apart’ are discriminated more quickly. The authors suggest that negative numbers are initially processed by children using rules, but that “symbolic manipulation can transform an existing magnitude representation so that it incorporates additional perceptual-motor structure.”

In summary, PMT suggests that learning how to perceptually and physically engage notations is critical to the capacity for reasoning in accordance with their mathematical meanings. To be successful, learners must discover which aspects of a notation are relevant and meaningfully aligned with mathematical rules and concepts, and must then acquire an appropriately “rigged up” sensorimotor system (see also: Goldstone et al., 2010). Although the sensorimotor skillset required for sophisticated symbolic reasoning is likely to be highly developed and available to learners only after some struggle (Piaget, 1953; Bednarz et al., 1996), Kellman et al. (2008) have already found that training students to recognize algebraic expressions using standard perceptual learning techniques leads to lasting gains both in equation reading and comprehension, as well as in algebraic problem-solving. Indeed, substantial evidence indicates that notation systems that align with computationally useful processes are relatively easy to acquire across a variety of domains including arithmetic and algebra (Kirshner and Awtry, 2004; Landy and Goldstone, 2007c), electric circuit design (Cheng, 1999), and sequence and grammar learning (Pothos et al., 2006; Endress et al., 2007). Our account expects such results because appropriate alignment between the formal and the perceptual significantly simplifies the search for correct solutions. Although we will not speculate extensively about possible implications for mathematics education, results such as these also suggest that the PMT approach can be a productive way to think about new pedagogical approaches to designing and reasoning with formal notations. In particular, it seems likely that the most effective and easily-learned notations and rule-systems are the ones that have greatest alignment with preexisting or easily learned perceptual and sensorimotor routines. On our view, one principal virtue of well-structured notation systems is that they leverage automatic sensorimotor operations by making their products formally useful, and the better the alignment between the formal and the sensorimotor, the more useful those products will be.

## Theoretical implications

### Is there a “fundamental” mathematical reasoning system?

A contribution of PMT is that it provides a novel account of how to bring mathematical and logical reasoning into the fold of embodied cognition more generally. Although PMT accommodates the cyborg view and its emphasis of the environment, it adds a detailed conception of the constitutive role of perceptual processing in symbolic reasoning: perception is at least as important as physical manipulation. One consequence of this view is that mathematical and logical reasoning need not be rooted in single, special-purpose cognitive mechanisms. Although we do not deny the existence of amodal numerosity or magnitude detection systems, our account does not assign those systems a uniquely fundamental role in the development of mathematical reasoning capacities. Instead, on our view symbolic reasoning is carried out by a wide variety of perceptual and motor skills, including fast numerosity and magnitude evaluation; repeatable actions like pointing, counting, and stacking; object segmentation and grouping; motion detection and visualization; writing and reading; and many other sensorimotor skills. Additionally, it seems reasonable to assume that the same sensorimotor skillset may also play a pivotal role in other mathematical domains such as geometry and category theory, the elementary portions of which both of which rely considerably on diagrams and other iconic notations. More controversially perhaps, since all areas of mathematics and symbolic reasoning involve—at some point—the learning of rules and abstract principles via notational systems, it may even be the case that the same perceptual and motor processes that implement the capacity for symbolic reasoning also play different but equally fundamental roles in implementing various kinds of abstract reasoning in mathematics and beyond. Whether this leaves any significant role for amodal systems remains to be seen, but see Dove (in press) for an argument for representational pluralism.

A corollary of the claim that symbolic and other forms of mathematical and logical reasoning are grounded in a wide variety of sensorimotor skills is that symbolic reasoning is likely to be both idiosyncratic and context-specific. For one, different individuals may rely on different embodied strategies, depending on their particular history of experience and engagement with particular notational systems. For another, even a single individual may rely on different strategies in different situations, depending on the particular notations being employed at the time. Some of the relevant strategies may cross modalities, and be applicable in various mathematical domains; others may exist only within a single modality and within a limited formal context. For example, consider the fact that there is significant potential for error when a successful strategy in one domain is exported to another domain—as, for example, when beginning logic students make the mistake of distributing a negation across a conjunction, going from ~(*X* & *Y*) to (~*X* & ~*Y*), because they perceive a similarity to the algebraically legal manipulation of −(*x* + *y*) to (−*x* + *y*). Although in this particular case such cross-domain mapping leads to a formal error, it need not always be mistaken—as when understanding that “~~*X*” is equivalent to “*X*,” just as “−−*x*” is equal to “*x*.” In some contexts, such perceptual strategies lead to mathematical success. In other contexts, however, the same strategies lead to mathematical failure.

If the capacity for symbolic reasoning is in fact idiosyncratic and context-dependent in the way suggested here, what are the implications for scientific psychology? PMT implies that the “deep” facts about human mathematical, algebraic, logical, and other mathematical abilities are unlikely to be facts about inner computations and models, but are instead facts about how humans manage to exploit perceptual and sensorimotor strategies in appropriate, context-specific ways—and about how they fall prey to these strategies when applying them inappropriately. The reason that mathematicians have the intuition that people who are merely “pushing symbols” are failing to grasp fundamental mathematical meanings is that they are indeed failing to do so—though this failure may be more widespread, and indeed more powerful, than mathematicians and psychologists have previously assumed. Being more specific than this, however, seems difficult. Therefore, the key to understanding the human capacity for symbolic reasoning in general will be to characterize *typical* sensorimotor strategies, and to understand the particular conditions in which those strategies are successful or unsuccessful.

### What is mathematical rule-following and who is the mathematical rule-follower?

Perceptual Manipulations Theory claims that symbolic reasoning is implemented over interactions between perceptual and motor processes with real or imagined notational environments. Since symbolic reasoning involves manipulating symbols and expressions according to mathematical and logical rules, this view implies that the human ability to follow *abstract* mathematical and logical rules is carried out by sensorimotor processes that apply to *concrete*—i.e., readily perceivable and physically manipulatable—notations. But how is it that “primitive” sensorimotor processes can give rise to some of the most sophisticated mathematical behaviors? Unlike many traditional accounts, PMT does not presuppose that mathematical and logical rules must be internally represented in order to be followed. Rather, overt rule-following emerges from the fine-tuned interactions between the perceptual and sensorimotor systems with well-designed physical notations—symbolic reasoning is a form of sophisticated “symbol pushing” that *happens* to adhere to the formal rules of mathematics and logic, due to a lengthy process of cultural adaptation and pedagogical scaffolding.

Like interlocking puzzle pieces that together form a larger image, sensorimotor mechanisms and physical notations “interlock” to produce sophisticated mathematical behaviors. Insofar as mathematical rule-following emerges from active engagement with physical notations, the mathematical rule-follower is a distributed system that spans the boundaries between brain, body, and environment. For this interlocking to promote mathematically appropriate behavior, however, the relevant perceptual and sensorimotor mechanisms must be just as well-trained as the physical notations must be well-designed. Thus, on one hand, the development of symbolic reasoning abilities in an individual subject will depend on the development of a sophisticated sensorimotor skillset in the way outlined above. On the other hand, the development of symbolic reasoning abilities within a society will depend on the availability of notational formalisms that promote formally valid “symbol-pushing.” Indeed, the development of mathematical expertise is often historically cotemporaneous with the development of powerful, efficient, and easily learned systems of formal mathematical and logical notation (Dantzig, 1954; Stedall, 2007).

## Conclusion

We have described an approach to symbolic reasoning which closely ties it to the perceptual and sensorimotor mechanisms that engage physical notations. We argued for this approach on the basis of empirical evidence that shows algebraic and mathematical knowledge to be surprisingly fragile in the face of minor perceivable differences, and on the basis of evidence that suggests that competent symbolic reasoners typically rely on semantically irrelevant properties of notational formulae in order to quickly and accurately—but also sometimes inaccurately—solve symbolic reasoning problems. With respect to this evidence, PMT compares favorably to traditional “translational” accounts of symbolic reasoning.

Nevertheless, there is probably no uniquely correct answer to the question of how people do mathematics. Indeed, it is important to consider the relative merits of all competing accounts and to incorporate the best elements of each. Just as the particular sensorimotor strategies being invoked are likely to differ across individuals and situations, it is also likely that different episodes of symbolic reasoning require different explanations—be they in terms of comparisons based on conceptual metaphors, situated interactions with notations, or even conscious applications of formal rules. Although we believe that most of our mathematical abilities are rooted in our past experience and engagement with notations, we do not depend on these notations at all times. Moreover, even when we do engage with physical notations, there is a place for semantic metaphors and conscious mathematical rule following. Therefore, although it seems likely that abstract mathematical ability relies heavily on personal histories of active engagement with notational formalisms, this is unlikely to be the story as a whole. It is also why non-human animals, despite in some cases having similar perceptual systems, fail to develop significant mathematical competence even when immersed in a human symbolic environment. Although some animals have been taught to order a small subset of the numerals (less than 10) and carry out simple numerosity tasks within that range, they fail to generalize the patterns required for the indefinite counting that children are capable of mastering, albeit with much time and effort. If we consider the working memory requirements for noticing that the pattern ___-ty one, ___-ty two, ___-ty three, etc. repeats after “twen-,” “thir-,” “for-,” and so on, then it may not seem so unlikely that only a species with a rather large brain could even notice let alone generalize the pattern. And without that basis for understanding the domain and range of symbols to which arithmetical operations can be applied, there is no basis for further development of mathematical competence.

Although we have not accounted for forms of mathematical reasoning beyond symbolic reasoning except in passing, the account of mathematical rule-following suggested here points toward the possibility that processes of perception, visualization, and interaction may play a crucial constitutive role in mathematical and logical reasoning in general. Unlike more established views, many of which acknowledge the utility of mathematical notations as concise representations of abstract mathematical meanings but then go on to downplay their importance for symbolic reasoning proper, PMT suggests that notations and the sensorimotor processes that engage them are often at the very heart of high-level mathematical and logical cognition. In this vein, since many forms of advanced mathematical reasoning rely on graphical representations and geometric principles, it would be surprising to find that perceptual and sensorimotor processes are *not* involved in a constitutive way. Therefore, by accounting for symbolic reasoning—perhaps the most abstract of all forms of mathematical reasoning—in perceptual and sensorimotor terms, we have attempted to lay the groundwork for an account of mathematical and logical reasoning more generally. The potential for a satisfying unification of the successes and failures of human symbolic and other forms of mathematical reasoning under a common set of mechanisms provides us with the confidence to claim that this is a topic worthy of further investigation, both empirical and philosophical.

### Conflict of interest statement

The authors declare that the research was conducted in the absence of any commercial or financial relationships that could be construed as a potential conflict of interest.
